# Epigenetic regulator G9a provides glucose as a sweet key to stress resistance

**DOI:** 10.1371/journal.pbio.3000236

**Published:** 2019-04-19

**Authors:** Kelsie R. S. Doering, Stefan Taubert

**Affiliations:** Centre for Molecular Medicine and Therapeutics, BC Children’s Hospital Research Institute, Department of Medical Genetics, The University of British Columbia, Vancouver, British Columbia, Canada

## Abstract

The ability to adapt to acute and chronic stress is important for organisms to thrive in evolutionary niches and for cells to survive in adverse conditions. The regulatory networks that control stress responses are evolutionarily conserved, and many factors that selectively activate stress responses have been identified. Less well understood are mechanisms that guard against unnecessary induction of cytoprotective factors and that connect stress responses with cellular metabolism to control energy expenditure during stress. The work of Riahi and colleagues represents important progress in this regard because it identifies the histone methyltransferase G9a as a modulator of oxidative stress responses. G9a dampens the expression of antioxidant genes, thus preventing inappropriate energy consumption. Moreover, G9a promotes the well-paced catabolism of storage glycogen and fat during stress. The importance of energy availability during stress is further evidenced by exogenous glucose rescuing the vulnerability of the G9a mutant to oxidative stress. Prior work in multiple model systems has implicated G9a in several other adaptive responses. Therefore, its role in pacing energy consumption and in restraining excessive stress response gene expression under stress may extend to other adaptive responses across species.

## Abstract 2

Stress responses are important for survival and evolutionary adaptation. This Primer explores a study showing that the fruit fly histone methyltransferase G9a (EHMT1/2) couples energy availability to finely tuned regulation of the stress response.

## Introduction

Throughout their lives, organisms, tissues, and cells may experience an abundance of stressors. These include suboptimal temperatures; mechanical stress or rupture; exposure to foreign, potentially toxic substances; nutrient or growth factor starvation; infection with pathogens; or an imbalance in the production of free radicals causing oxidative stress. The ability to mount tailor-made responses that promote short-term survival and long-term adaptation to such insults is critical for cell survival in a multicellular entity and for evolutionary niche adaptation of organisms. Investigating these responses at the whole-organism level in the context of evolution and ecology has been especially insightful because stressors and corresponding adaptive responses can determine the distribution of a species [1] and may even contribute to the evolution of new cell types [2]. In addition, cellular stressors and stress responses are of enormous pathophysiological significance. Many of the most prevalent human diseases feature cellular stress as contributing, exacerbating, or triggering factors. For example, a hallmark of cancer cells is their ability to survive and thrive in the face of stressors such as starvation, oxidative stress, hypoxia, low pH, and others [3–5]. Stress resistance is essential in solid tumors with uncontrolled cell division and inadequate vascularization and in circulating cancer cells and metastases, which are exposed to hostile microenvironments [6,7]. Cancer cells therefore hijack stress resistance pathways, and targeting the underlying factors promoting this adaption is a promising strategy to treat cancers [8].

Over the last 2 decades, powerful omics technologies have enabled first the cataloging of genes and proteins that respond to certain stressors. In turn, this led to the identification of regulatory factors and pathways that orchestrate these responses. Collectively, these studies have revealed intricate response networks coordinating transcriptional and translational changes to implement molecular and cellular adaptation [9,10]. Such responses include stressor-specific defenses, such as the induction of innate immune responses and xenobiotic detoxification enzymes; broader mechanisms that sense and repair macromolecule (nucleic acid, protein, and lipid) damage no matter the type of original insult; and efforts towards maintaining cellular homeostasis by altering energy metabolism and controlling cell growth, proliferation, and differentiation. Should the accumulated damage be too severe, cells may choose to undergo programmed cell death (apoptosis) [9,11].

An important stress all organisms face is oxidative stress, which is caused when reactive oxygen species (ROS), obligate and ubiquitous byproducts of aerobic respiration, accumulate in cells to toxic levels. ROS are highly electrophilic and cause cellular damage by oxidizing DNA, proteins, and lipids. However, at lower, controlled levels, ROS enable physiological processes such as immune responses and development [12,13]. Therefore, ROS levels must be properly controlled to ensure adequate function while avoiding damage. To date, many evolutionarily conserved “master regulators” of stress responses have been identified. Often, these are transcription factors necessary and sufficient to drive individual stress responses [10], including the response to excess ROS [14]. Less well understood are mechanisms that terminate these responses once the insult is no longer acute or that temper excessive gene activation in both ambient and stressful conditions. Inadequate expression of cytoprotective genes is not only potentially harmful but also energetically wasteful. Therefore, elucidating the mechanisms by which cells ensure adequate expression of these genes and connecting them to the regulation of energy metabolism is of great interest.

**Fig 1:**
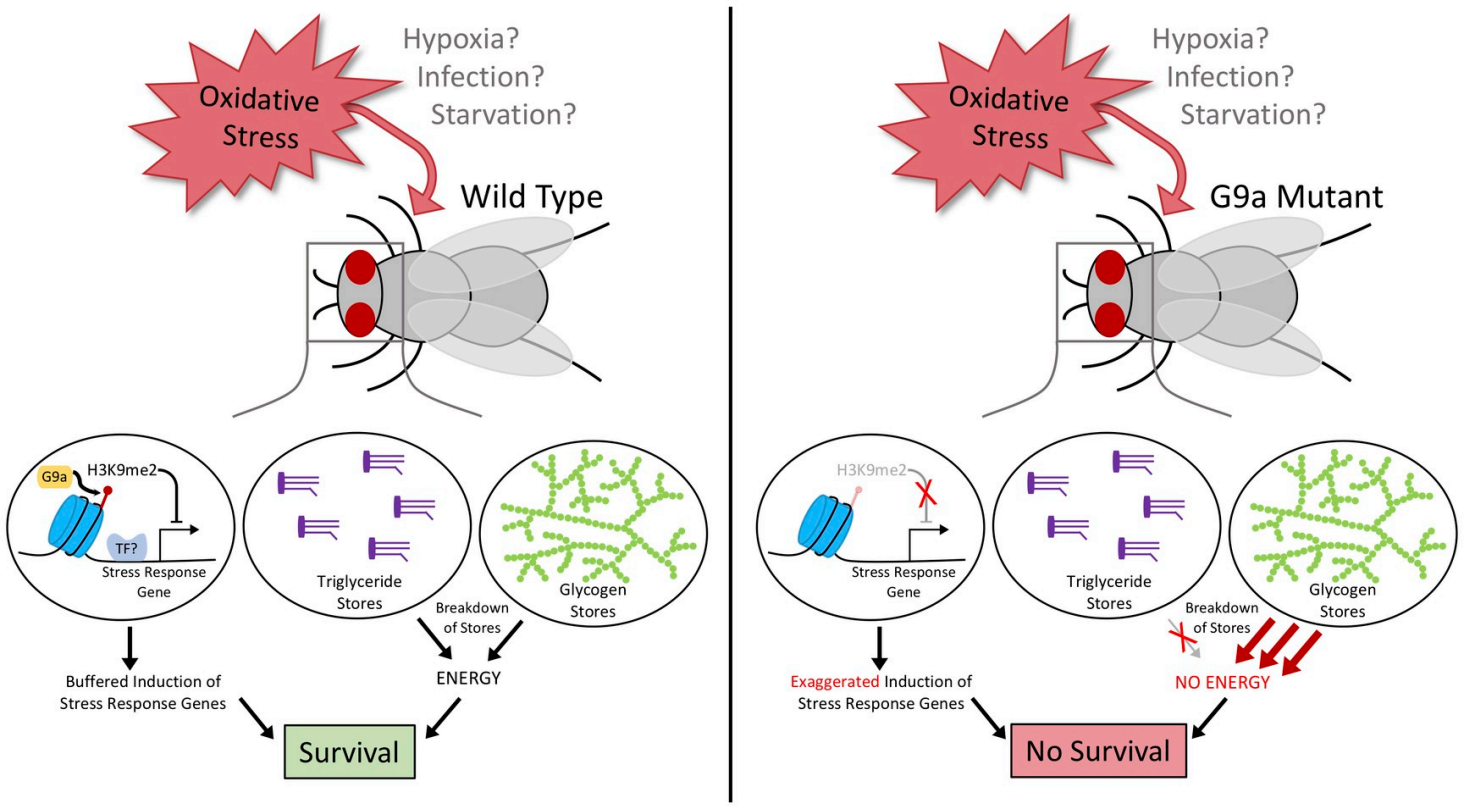
Schematic representation of G9a dependent stress protection. In wild-type flies (left), G9a promotes H3K9 dimethylation. This protects flies from oxidative stress by dampening the expression of cytoprotective genes such as catalases and by allowing coordinated access to stored energy (triglycerides and glycogen). G9a may promote similar responses in other harmful conditions, such as in hypoxia, viral or other pathogenic infection, or in starvation. In G9a mutants (right), these responses are compromised: stress response genes are hyperactivated, lipid catabolism is blocked, and glycogen is wasted. The sum of these effects leads to reduced survival in oxidative stress. H3K9me2, histone H3 lysine 9 dimethylation; TF, transcription factor.

In this issue of *PLOS Biology*, Riahi and colleagues show that the histone methyltransferase G9a (the common ancestor of mammalian Euchromatic histone-lysine N-methyltransferases 1 and 2 [EHMT1 and EHMT2]) protects *Drosophila melanogaster* from oxidative stress by dampening the excessive activation of antioxidant genes and by promoting access to metabolic energy [15]. The authors and others have previously described a role for *D*. *melanogaster* G9a in the response to viral infection and starvation. Additional reports demonstrated requirements for G9a in metabolic and hypoxia adaptation in mice and in mammalian cell culture [16–19]. In these studies, G9a influenced survival by regulating the expression of specific stress response pathways, such as signaling through the Janus kinase-Signal Transducer and Activator of Transcription (Jak-STAT) innate immune response pathway, the hypoxia response master regulator hypoxia inducible factor 1 (HIF-1), or the cytoprotective Phosphoinositide 3-kinase/Protein kinase B/Nuclear factor (erythroid-derived 2)-like 2 (PI3K/PKB/Nrf2) axis. In the present study, the authors used the herbicide paraquat, a pro-oxidant that converts oxygen to superoxide, to show that G9a is required for animal population survival in oxidative stress. To delineate how G9a rewires genome transcription in oxidative stress, they profiled transcriptomes by RNA-sequencing (RNA-seq). G9a mutant flies hyperactivated genes involved in the response to oxidative stress. In particular, genes encoding catalases and peroxiredoxins, which reduce hydrogen peroxide to water and are vital for oxidative stress resistance [12], were strongly induced in G9a mutants. Previously, the authors had used chromatin immunoprecipitation sequencing (ChIP-seq) to profile genes that bear histone H3 lysine 9 dimethylation (H3K9me2) chromatin marks, which are deposited by G9a, in whole larvae [20]. Among others, this identified catalase and peroxiredoxin genes, suggesting that these are direct regulatory targets for G9a. Based on these data, the authors proposed that G9a represses oxidative stress-induced antioxidant genes and thus “buffers” against an exaggerated transcriptional response (Fig 1).

Digging deeper, the RNA-seq data revealed that stressed G9a mutants failed to activate genes involved in cellular metabolic processes. Therefore, the authors postulated that G9a mutants were unable to use potentially available energy. Indeed, unlike control flies, G9a mutants failed to mobilize storage triglycerides from the head fat body following oxidative stress. Next, the authors assessed the rate of glycogen use. G9a mutants stored much higher amounts of glycogen than controls at steady state, but when exposed to oxidative stress, they completely depleted all stored glycogen in only 24 hours. Notably, this timeframe coincided tightly with the average time of death of G9a mutants. This suggested that glycogen depletion is a central contributor to the oxidative stress sensitivity of G9a mutant flies. Indeed, feeding the mutants a high glucose diet was sufficient to substantially extend their life span in oxidative stress. Moreover, directly limiting glycogen-to-glucose breakdown by mutating the glycogen phosphorylase (GlyP) gene phenocopied the oxidative stress sensitivity of G9a mutants. Lastly, control flies fed a high glucose diet also showed increased survival on oxidative stress. These data pinpoint rapid energy access as an important factor in limiting oxidative stress sensitivity, at least in flies. Collectively, these experiments show that G9a mutant flies are sensitive to oxidative stress because they have a hyperactive, energy-consuming oxidative stress response and are unable to effectively access their lipid stores while wasting their glycogen deposits (Fig 1).

These findings are interesting because they suggest that G9a is a core component of a mechanism that connects the regulation of specific stress response genes (here oxidative stress response genes) to the control of energy metabolism. This regulatory pathway may be relevant in multiple adverse conditions, because G9a has been implicated in several different stress responses across species ([16–19] and Figure S10 in [15]). The key roles of G9a in mediating these responses may be assuring adequate energy allocation during times of stress as well as mediating a buffered induction of specific stress response gene sets.

Expanding on the elegant work by Riahi and colleagues, future studies should test the idea that metabolic defects similar to those identified here may generally contribute to stress sensitivity and that limited energy availability may underlie harmful effects of stress even without overt metabolic defects. As such, rapidly accessible energy reserves or simple provision of dietary glucose may promote survival in various stressors across species. Glycogen catabolism mediated by G9a could be of particular importance in hypoxia (when fat catabolism by β-oxidation is attenuated) and in starvation (wherein glycogen is catabolized before fat stores are accessed). In *Caenorhabditis elegans*, access to glycogen stores confers oxidative stress resistance by promoting nicotinamide adenine dinucleotide phosphate (NADPH) and/or glutathione reduction and mediates hyperosmotic stress resistance by providing glucose for conversion into the organic osmolyte glycerol [21,22]. Although such glycogen and/or glucose utilization appears quite stress specific, it nevertheless highlights the versatile importance of energy stores in diverse stressors. Ultimately, the role of G9a in stress response regulation is likely complex, integrating stress-specific roles such as altering innate immunity by modulating Jak-STAT signaling [17] with the broadly relevant modulation of energy metabolism (Fig 1).

In stark contrast to the rapid glycogen wasting was the complete inability of G9a mutant flies to access stored fat. In wild-type flies, triglyceride breakdown appeared to follow slower kinetics, perhaps because oxidative fat catabolism is undesirable because it may itself generate ROS. Similar considerations apply to hypoxia, wherein cells and organisms cannot use the substantial energy stored in triglycerides. Nevertheless, accessing the energy stored in fat could be especially important during prolonged stresses, and recent studies in *C*. *elegans* show that transcriptional regulators of fatty acid catabolism are also vital for stress resistance and vice versa [23–25]. Future investigations, perhaps using *Drosophila* mutants defective in specific lipid catabolism enzymes, could pinpoint whether triglycerides indeed provide essential energy to fend off, e.g., sustained infections.

Another intriguing finding by Riahi and colleagues is that G9a mutants deregulate a cluster of genes implicated in sensory perception and cilia function [15]. This is noteworthy because, in starvation, G9a modulates the expression of gustatory receptors that sense sugar, and loss of G9a leads to altered sensitivity and locomotion [16]. As such, it is possible that G9a regulation of a similar gene set controls fly behavior when oxidative or other stressors are detected—avoidance or escape are, after all, highly effective defences for mobile species [26].

Going forward, it will also be interesting to more precisely delineate the mechanism of gene regulation by G9a in various adverse conditions. Integrating the knowledge gained from studies on *C*. *elegans* glycogen mobilization [21,22], it would appear reasonable to investigate adenosine monophosphate (AMP) -activated protein kinase (AMPK) as an upstream regulator of G9a. Moreover, recruitment of G9a to chromatin is likely mediated by stress-activated transcription factor(s). Identifying such proteins and the signaling pathways through which they cooperate with G9a would provide important mechanistic insights.

Another area for further study is an investigation of a potential sex-specific role for G9a in metabolic control. In *Drosophila*, sex influences the expression of antioxidant genes, and sex-specific protease expression and proteolytic activity confer selective resistance to different types of oxidative stress [27,28]. In the present study, Riahi and colleagues appeared to investigate male flies exclusively [15]. However, their prior work showed that the hypersensitivity of G9a mutants to virus infection was not sex dependent [17]. Future work to test whether energy access and stress gene modulation by chromatin regulation via G9a is sex specific would be illuminating. This is especially compelling because energy metabolism, identified here as a reason underlying the stress sensitivity in G9a mutants, differs substantially between sexes. Viewed more broadly, sex-driven differences in such fundamentally important processes could be relevant in human pathophysiological contexts, because women have a reduced mortality risk from several chronic diseases. Exploring these questions will undoubtedly be an important endeavour in future years.

## Abbreviations

AMP: adenosine monophosphate
AMPK: 5' AMP-activated protein kinase
ChIP-seq: chromatin immunoprecipitation sequencing
EHMT2: Euchromatic histone-lysine N-methyltransferase 2
GlyP: glycogen phosphorylase
H3K9me2: histone H3 lysine 9 dimethylation
HIF-1: hypoxia inducible factor 1
Jak-STAT: Janus kinases-signal transducer and activator of transcription proteins
NADPH: nicotinamide adenine dinucleotide phosphate
Nrf2: Nuclear factor (erythroid-derived 2)-like 2
PI3K: Phosphoinositide 3-kinase
PKB: Protein kinase B
RNA-seq: RNA-sequencing
ROS: reactive oxygen species
